# Displacement of the Dilated Stomach

**Published:** 1900-06-09

**Authors:** 


### DISPLACEMENT OF THE DILATED STOMACH.

IN a clinical lecture recently delivered at Guy's Hospital, Dr. Taylor referred to the great amount of displacement which the stomach might undergo when it became dilated in consequence of disease of the pylorus. Normally, he said, the pylorus lies very close to the middle line, and the lower border of the stomach projects not very far below the costal margin; the left lobe of the liver lies across the epigastrium, and the stomach comes a little below it, but varies, of course, with conditions of varying physiological distension. When, however, there is obstruction, and consequent dilatation, from a pyloric cancer, the enlargement of the stomach extends downwards towards the umbilicus, and when the dilatation is extreme, while the lower border may extend below the umbilicus, the epigastric region may be flat, corresponding to a space above the displaced stomach. But the pylorus itself may be much displaced, and in illustration of this we mentioned a case in which an enlarged pylorus was situated in the right iliac fossa, and had the dilated stomach vertically above it.

